# The mechanosensitive ion channel ASIC2 mediates both proprioceptive sensing and spinal alignment

**DOI:** 10.1113/EP090776

**Published:** 2023-03-23

**Authors:** Bavat Bornstein, Bridgette Watkins, Fabian S. Passini, Ronen Blecher, Eran Assaraf, Xiao Meng Sui, Vlad Brumfeld, Michael Tsoory, Stephan Kröger, Elazar Zelzer

**Affiliations:** ^1^ Department of Molecular Genetics Weizmann Institute of Science Rehovot Israel; ^2^ Department of Physiological Genomics, Biomedical Center Ludwig‐Maximilians‐University Planegg‐Martinsried Germany; ^3^ Orthopedic Department Assuta Ashdod University Hospital, Ashdod, Israel, affiliated to Ben Gurion University of the Negev Beer Sheba Israel; ^4^ Department of Orthopedic Surgery Shamir Medical Center, Assaf HaRofeh Campus, Zeffifin, Israel, affiliated to Sackler Faculty of Medicine, Tel Aviv University Tel Aviv Israel; ^5^ Department of Chemical Research Support Weizmann Institute of Science Rehovot Israel; ^6^ Department of Veterinary Resources Weizmann Institute of Science Rehovot Israel

**Keywords:** ASICs, ion channel, mechanosensitivity, muscle spindle, proprioception, scoliosis

## Abstract

By translating mechanical forces into molecular signals, proprioceptive neurons provide the CNS with information on muscle length and tension, which is necessary to control posture and movement. However, the identities of the molecular players that mediate proprioceptive sensing are largely unknown. Here, we confirm the expression of the mechanosensitive ion channel ASIC2 in proprioceptive sensory neurons. By combining in vivo proprioception‐related functional tests with ex vivo electrophysiological analyses of muscle spindles, we showed that mice lacking *Asic2* display impairments in muscle spindle responses to stretch and motor coordination tasks. Finally, analysis of skeletons of *Asic2* loss‐of‐function mice revealed a specific effect on spinal alignment. Overall, we identify ASIC2 as a key component in proprioceptive sensing and a regulator of spine alignment.

## Abstract (highlights)

- **What is the central question of this study?**
  Proprioception is initiated by mechanosensitive neurons. However, the identities of the molecular players that mediate proprioceptive sensing are largely unknown. Here, we aimed to identify potential mechanosensitive ion channels that mediate proprioceptive signalling.
- **What is the main finding and its importance?**
  We identify the mechanosensitive ion channel ASIC2 as a key component in proprioceptive sensing and a regulator of spine alignment.

## 1 INTRODUCTION

Proprioception is the sense of self‐movement and body position and, thus, it is essential for controlling coordinated movements and posture (Sherrington, 1907). In mammals, proprioception is initiated by mechanically sensitive sensory neurons whose cell bodies are located in the dorsal root ganglia (DRGs). These neurons project peripherally to muscles and tendons, ending in two types of sensory organs: muscle spindles (MSs), which lie in the muscle belly; and Golgi tendon organs (GTOs), which are located at the muscle–tendon interface. Proprioceptive neurons terminate in different locations in the CNS. They project into the ventral horn of the spinal cord onto α‐motor neurons or interneurons to influence premotor circuits (Chen et al., 2003; Delhaye et al., 2018). Additionally, they ascend via the dorsal column tract to synapse onto neurons in the dorsal column nuclei of the brainstem to relay information on the tensions and forces acting on the muscles (Delhaye et al., 2018; Kiehn, 2016; Marasco & de Nooij, 2022).

Recently, the proprioceptive system has also been shown to regulate skeletal development and function, specifically spinal alignment, bone fracture repair and joint morphogenesis (Assaraf et al., 2020; Blecher, Krief, Galili, Assaraf, et al., 2017; Blecher, Krief, Galili, Biton, et al., 2017; Bornstein et al., 2021). The importance of the proprioceptive system for controlling coordination and posture, in addition to its involvement in the aetiology of skeletal pathologies, emphasize the need to understand the molecular mechanisms underlying its function. However, the available information about these mechanisms is limited. Only in recent years have transcriptional analyses of DRG proprioceptive neurons (Oliver et al., 2021; Wu et al., 2021) and of MSs (Bornstein et al., 2023; Kim et al., 2020) provided the opportunity to find new molecules that mediate proprioceptive sensing and regulatory functions.

To date, two mechanosensitive ion channels have been implicated in mechanotransduction of proprioceptive sensory neurons (Wilkinson, 2022). *Piezo2*, which encodes a calcium‐permeable ion channel, was shown to be expressed in sensory endings in MSs and GTOs (Woo et al., 2015). Its ablation resulted in loss of mechanotransduction in the proprioceptive neurons, in addition to scoliosis and hip dysplasia (Assaraf et al., 2020; Chesler AT et al., 2016; Woo et al., 2015). The other channel is acid‐sensing ion channel 3 (ASIC3), a member of the ASIC family of proton‐gated cation channels found in the central and peripheral nervous systems. *Asic3* loss of function in mice impaired mechanotransduction in proprioceptive neurons and led to deficits in motor tasks (Lin et al., 2016). Upon activation, the ASIC complex, composed of three homotrimeric and/or heterotrimeric subunits, induces neuronal depolarization via Na^+^ ion influx. Although ASICs were originally shown to mediate acid sensing, they can also act as mechanically activated ion channels and, thereby, transform mechanical force into an electrical signal (Cheng et al., 2018). In mice, six ASIC isoforms are encoded by four genes. However, only ASIC1, ASIC2 and ASIC3 were shown to be expressed in DRG proprioceptive neurons (Lin et al., 2016; Wu et al., 2021), and only ASIC2 and ASIC3 were shown to be expressed in proprioceptive neuron terminals (Lin et al., 2016; Simon et al., 2010). Interestingly, amiloride, which antagonizes epithelial sodium channels such as ASICs, was shown to inhibit muscle spindle afferent discharge (Simon et al., 2010), raising the question of whether other ASICs besides ASIC3 contribute to proprioceptive mechanosensing.

To identify molecules that are important for proprioceptive sensing, we recently generated comprehensive transcriptomic and proteomic datasets of the entire MS (Bornstein et al., 2023). Here, we studied the involvement of *Asic2*, which was the most differentially expressed mechanosensitive ion channel in our transcriptomic data, in proprioceptive function and skeletal regulation. We detected ASIC2 protein expression in proprioceptive neuron endings of both MSs and GTOs. Moreover, *Asic2* knockout (KO) in mice showed that ASIC2 mediates proprioceptive sensing, coordinated movement and spinal alignment. These findings implicate ASIC2 in proprioceptive mechanotransduction and skeletal alignment and reinforce the importance of regulatory interactions between the proprioceptive system and the skeleton.

## 2 METHODS

### 2.1 Ethical approval

All experiments involving mice were approved by the institutional animal care and use committee of the Weizmann Institute (protocol number #02190222‐2). Electrophysiology experiments were performed according to Directive 2010/63/EU of the European Parliament on the protection of animals used for scientific purposes and were approved by the local authorities of the State of Bavaria, Germany (Az.: ROB‐55.2‐2532.Vet 02‐17‐82). In all cases, experimental protocols were designed to minimize the number of animals used.

### 2.2 Mouse lines

Mice were housed in a temperature‐ and humidity‐controlled vivarium on a 12 h–12 h light–dark cycle with free access to food and water.

The following strains were used: *Asic2* KO (*Asic2^tm1Wsh^*/J; The Jackson Laboratory; #013126), *Thy1‐YFP16* (The Jackson Laboratory; #003709), *Pvalb^Cre^* (*Pvalb^tm1(cre)Arbr^*/J; The Jackson Laboratory; #017320) and *Rosa26^tdTomato^* (The Jackson Laboratory, #007909). In all experiments, *Asic2* KO mice are *Asic2* ^−/−^ and control mice are littermates that are either *Asic2* ^+/+^ or *Asic2* ^−/+^. Mice were genotyped by PCR of genomic DNA from ear clips. Primer sequences and amplicon sizes are listed in Table 1.

**TABLE 1:** Primer sequences and amplicon sizes used for PCR.

**Reaction**	**Amplicon (bp)**	**Sequence**
YFP (GFP)	300	Forward: GACGGCAACATCCTGGGGCACAAG Reverse: CGGCGGCGGTCACGAACTCC
*Asic2* knockout	Wild‐type: 365 Mutant: 300	Wild‐type forward: GAAGAGGAAGGGAGCCATGATGAG Mutant forward: TGGATGTGGAATGTGTGCGA Common reverse: AGTCCTGCACGGTGGGAGCTTCTA
Cre	800	Forward: CCTGGAAAATGCTTCTGTCCGTTTGCC Reverse: GAGTTGATAGCTGGCTGGTGGCAGATG
tdTomato (wild‐type)	297	Forward: AAGGGAGCTGCAGTGGAGTA Reverse: CCGAAAATCTGTGGGAAGTC
tdTomato (floxed allele)	196	Forward: GGCATTAAAGCAGCGTATCC Reverse: CTGTTCCTGTACGGCATGG

### 2.3 Immunofluorescence

For whole‐mount immunofluorescence, muscles were subjected to an optical tissue clearing protocol as described previously (Bornstein et al., 2023). Briefly, postfixed deep masseter muscle or extensor digitorum longus (EDL) muscle was dissected, washed in PBS and placed in A4P0 hydrogel {4% acrylamide and 0.25% 2′‐azobis[2‐(2‐imidazolin‐2‐yl)propane]dihydrochloride in PBS}, shaking at 4°C overnight. Then the hydrogel was allowed to polymerize for 3 h at 37°C. Next, samples were washed in PBS, transferred to 5 mL of 10% SDS (pH 8.0) with 0.01% sodium azide, and shaken gently at 37°C for 3 days to remove lipid.

Cleared samples were washed with wash buffer (PBS containing 0.5% Tween‐20) for 20 min, permeabilized with PBST (PBS containing 0.3% Triton X‐100) for 20 min and washed again with wash buffer for 20 min, all at room temperature with shaking. Then the samples were blocked with 6% bovine serum albumin dissolved in PBS containing 0.3% Triton X‐100 and 0.5% Tween‐20 for 2 days at 37°C, with gentle shaking. Samples were subjected to primary antibodies (Table 2) for 5 days at 37°C, with gentle shaking, washed with wash buffer for 2 days at room temperature with frequent solution changes, incubated with secondary antibodies and 4′,6‐diamidino‐2‐phenylindole (DAPI) for 5 days at 37°C, with shaking, and washed again with wash buffer for 2 days at room temperature, with frequent solution changes. For clearing and mounting, the samples were incubated in 500 µL of refractive index matching solution (RIMS; 74% w/v Histodenz in 0.02 M phosphate buffer) for 1 day at room temperature, with shaking. Samples were mounted in RIMS and imaged using a Zeiss LSM800 or LSM900 confocal microscope. Images were processed with ImageJ v.1.51 (US National Institutes of Health).

**TABLE 2:** Antibodies used for immunofluorescence.

**Target**	**Species**	**Company**	**Catalogue number**	**Dilution**
GFP	Goat	Abcam	Ab6658	1:100
ASIC2	Rabbit	LSBio	LS‑B156	1:100
GLUT1	Rabbit	Abcam	ab195020	1:400
Rabbit	Cy5 conjugated donkey	Jackson Immunoresearch Laboratories	711‐175‐152	1:200
Biotin	Native streptavidin protein (DyLight 488)	Abcam	b134349	1:200

For DRG immunofluorescence, DRGs were isolated as described previously (Sleigh et al., 2016), fixed for 3 h in 4% paraformaldehyde (PFA)/PBS at 4°C, transferred to 30% sucrose overnight, then embedded in optimal cutting temperature (OCT) compound and sectioned by cryostat at a thickness of 10 µm. For ASIC2 staining, cryosections were dried and post‐fixed for 10 min in 4% PFA, permeabilized with PBS with 0.3% Triton X‐100, washed with PBS with 0.1% Tween‐20 (PBST) for 5 min and blocked with 7% goat/horse serum and 1% bovine serum albumin dissolved in PBST. Then sections were incubated with primary antibody (Table 2) at 4°C overnight. The next day, sections were washed three times in PBST and incubated for 1 h with secondary antibody‐conjugated fluorescent antibody, washed three times in PBST, counterstained with DAPI, mounted with Immu‐Mount aqueous‐based mounting medium (Thermo Fisher Scientific) and imaged using a Zeiss LSM800 or LSM900 confocal microscope. Images were processed with ImageJ v.1.51 (US National Institutes of Health).

### 2.4 Behavioural procedures

Behavioural tests were performed on adult males (>90 days old) during the dark phase of the circadian cycle after ≥1 h of habituation to the test room, unless stated otherwise.

#### 2.4.1 Beam walking

Males were first trained to walk on a beam (50 cm long, 35 mm wide) suspended 30 cm above the working surface, in order to return to their home cage. Then the mice were tested by walking on a narrow beam (6 mm wide) to return to their home cages. Five full‐length walks with no stops were counted. Test sessions were video recorded using an overhead camera. The number of slips and travel time during the walks were measured for *Asic2* KO mice and their littermate controls.

#### 2.4.2 Home cage locomotion

Locomotion was assessed using the InfraMot system (TSE Systems). Mice were housed individually for 72 h, during which the first 24 h were considered habituation to the individual housing conditions. Measurements of locomotion was collected in 30 min intervals. General locomotion was measured as the mean of two light and two dark cycles during the last 48 h.

#### 2.4.3 Treadmill

The treadmill apparatus (Panlab; Harvard Apparatus; LE8710M) consisted of a rolling belt with adjustable speed and acceleration, with a grid situated at the end of the rolling belt to provide an electrical shock. Mice were tested on a 2‐day protocol consisting of a habituation day, followed by a test day. During the habituation day, mice were placed on the treadmill for 10 min with the shocker operating at 0.2 mA while the treadmill belt was not moving. During the test day, mice were subjected to 15 min treadmill acceleration according to a crescendo protocol. Mice started with 10 min of gradual speed increments from 0.1 to 0.18 m/h, followed by 5 min of speed increment from 0.18 to 0.19 m/h. The total distance the mice travelled in 15 min was measured.

#### 2.4.4 CatWalk

Gait was assessed using the CatWalk XT 10.6 automated gait analysis system (Noldus Information Technology, Wageningen, The Netherlands). For each mouse, five runs were recorded. A successive run was determined by a duration range of 2–10 s and maximum variation of 60%. After the identification and labelling of each footprint, gait data were generated. The following parameters were analysed for each mouse: mean speed, phase dispersion, step sequence, stride length, print position and support.

### 2.5 Electrophysiology

#### 2.5.1 Animals

Experiments were performed on muscles from nine C57BL/6J mice, including three heterozygous and four homozygous *Asic2* KO and two wild‐type (WT) mice of both sexes. Age ranged between 10 and 15 weeks and weight between 22 and 28 g.

#### 2.5.2 Muscle preparation and electrophysiology

Afferent sensory neuron responses to stretch were assayed using an isolated muscle–nerve preparation previously described (Franco et al., 2014; Gerwin et al., 2019, 2020; Wilkinson et al., 2012). Twenty‐four MSs from control animals (heterozygous and WT mice) and 21 muscle spindles from homozygous *Asic2* KO mice were recorded. Mice were killed by cervical dislocation to avoid interference of anaesthetic agent with the sensory afferent recordings. The EDL muscle together with the deep peroneal branch of the sciatic nerve were dissected and placed in a tissue bath containing oxygenated artificial cerebrospinal fluid (Wilkinson et al., 2012). The tendons were sutured at one end to a fixed post and at the other end to a lever arm connected to a dual force and length controller (300C‐LR; Aurora Scientific, Dublin, Ireland), allowing the simultaneous recording of muscle tension and muscle length. Sensory activity was sampled using a suction electrode (tip diameter, 50−70 µm) connected to an extracellular amplifier (model 1800, A&M Systems, Elkhart, USA). The standard solution for muscle spindle afferent recordings was oxygenated artificial cerebrospinal fluid containing (mM): 128 NaCl, 1.9 KCl, 2.4 CaCl_2_, 1.3 MgSO_4_, 1.2 KH_2_PO_4_, 26 NaHCO_3_ and 10 d‐glucose.

A signal was classified as being from a putative muscle spindle afferent if it displayed a characteristic instantaneous frequency response to stretch, in addition to a pause during twitch contraction (Franco et al., 2014; Gerwin et al., 2019; Wilkinson et al., 2012). Baseline muscle length (*L*_0_) was defined as the minimal length at which maximal twitch contractile force was generated. For each recording, triplicates of 10 s resting discharge followed by ramp‐and‐hold stretches (*L*_0_ plus 2.5, 5.0 and 7.5% of *L*_0_; ramp speed, 40% *L*_0_/s; ramp phase duration, 0.1 s; hold phase, 3.8 s; stretch duration, 4 s with 45 s intervals between each stretch; Gerwin et al., 2020) were recorded and averaged.

From these recordings, the mean resting discharge (RD; mean baseline firing rate) and the dynamic peak (DP; highest firing rate during ramp minus baseline firing rate), the dynamic index (DI; dynamic peak minus firing rate 0.45−0.55 s into stretch minus baseline firing rate), the initial static time (IST; dynamic peak minus firing rate 0.45−0.55 s into stretch minus baseline firing rate) and the final static time (FST; firing rate 3.25−3.75 s into stretch minus baseline firing rate) were determined (Gerwin et al., 2019; Kröger & Watkins, 2021).

For data analysis, action potentials from individual sensory neurons were identified by spike shape and spike discriminator using the Spike Histogram feature of LabChart (v.8.1.5; AD Instruments, Sydney, NSW, Australia). Action potentials from additional potential muscle spindles that appeared during the stretch, detectable by different frequencies and amplitudes, were not scored. No attempt was made to discriminate group Ia afferents from group II (for a detailed discussion, see Wilkinson et al., 2012).

#### 2.5.3 Maximal tetanic force

At the end of each recording, the maximal contractile force during a direct tetanic stimulation of the muscle was determined as previously described (Gerwin et al., 2019, 2020; Wilkinson et al., 2012). Muscles were stimulated via paddle electrodes in the tissue bath (500 ms train at 120 Hz and ∼1 ms pulse length, supramaximal voltage; Grass SD9 stimulator; Natus, Pleasanton, CA, USA). The specific force (force/cross‐sectional area) of the EDL muscle at *L*_0_, which is a measure of the general health status of the muscle, was determined and compared with the previously reported peak force of a healthy EDL of 23.466 ± 6 N/cm^2^ (Brooks & Faulkner, 1988; Larsson & Edström, 1986).

The instantaneous frequency [in impulses per second (imp/sec)] was compared between control and *Asic2* KO mice. Only single‐unit spindle afferent responses that could be recorded without interruption throughout the entire experiment were included in the analysis. For the ramp‐and‐hold stretches, baseline values for all parameters (baseline firing rate, DP, IST and FST) were determined as a mean of three stretches, and the mean of all recordings was compared between WT and mutant mice. Values are reported as mean of the frequency (in impulses per second) in a dot plot, with each dot representing a different muscle spindle response.

### 2.6 In vivo micro‐computed tomography

In vivo micro‐CT scans were performed on adult male and female mice (>90 days old) using the SkyScan 1276 system (Bruker). Before scanning, mice were anaesthetized by inhalation of isoflurane using inhalation chamber, with maintenance by inhalation mask during the scan. The entire spine was scanned in a continuous rotation mode, with a scanning speed of 40 s (360 rotations). Owing to the length limit, imaging was occasionally performed in three overlapping parts that were then merged into one dataset representing the entire region of interest. The total radiation dose was ∼500 mGy. All micro‐CT scans were reconstructed using N‐recon software. Three‐dimensional volume rendering images were produced using Amira software (Thermo Fisher Scientific).

### 2.7 Measurements of spinal deformity

To measure the spinal curves of *Asic2* KO and control mice, we calculated the Cobb angle (Cobb, 1948) on three‐dimensionally rendered images of the micro‐CT scans, as described previously (Blecher, Krief, Galili, Assaraf et al., 2017). For scoliosis, the vertebrae that were the most side‐tilted rostrally and caudally in the coronal plane were identified. Then, the angle between a line parallel to the superior endplate of the rostral end vertebra and a line parallel to the inferior endplate of the caudal end vertebra was measured. For kyphosis, the Cobb angle was measured between lines parallel to the superior and inferior end vertebrae in the sagittal plane.

### 2.8 Ex vivo micro‐computed tomography

Ex vivo micro‐CT scans of the hip joint were performed on male and female mice (>180 days old) using an Xradia MicroXCT‐400 scanner. Tissue was fixed overnight in 4% PFA–PBS and dehydrated to 100% ethanol. The source was set at 40 kV and 200 µA. A total of 1500 projections were taken over 180° with an exposure time of 1.5 s. The final voxel size was 10 µm.

### 2.9 Measurements of hip deformity

Hip morphology was analysed on ex vivo hip joint CT scans using Amira software (Thermo Fisher Scientific). The acetabular index and congruency index were measured on a coronal slice of the hip joint, as described before (Assaraf et al., 2020). The acetabular index was measured between a horizontal line connecting both acetabular centres and a line extending from the acetabular centre to the sourcil. The congruency index was calculated for the upper acetabular roof as the mean distance along the joint line divided by the minimal value out of all measurements. A value of one indicates perfect congruence, whereas incongruence results in higher values.

### 2.10 Statistical analysis

Statistical significance was determined using Student's unpaired two‐tailed *t*‐test when comparing two samples, whereas one‐way or two‐way ANOVA was used to compare multiple samples. All statistical analyses were performed using GraphPad Prism (v.9.3; Graphpad Software, La Jolla, CA, USA). For all statistical tests, significance was defined as a *P*‐value <0.05. A summary of the statistical test used is provided in Supplementary File.

## 3 RESULTS

### 3.1 *Asic2* is expressed in proprioceptive neurons

**FIGURE 1:**
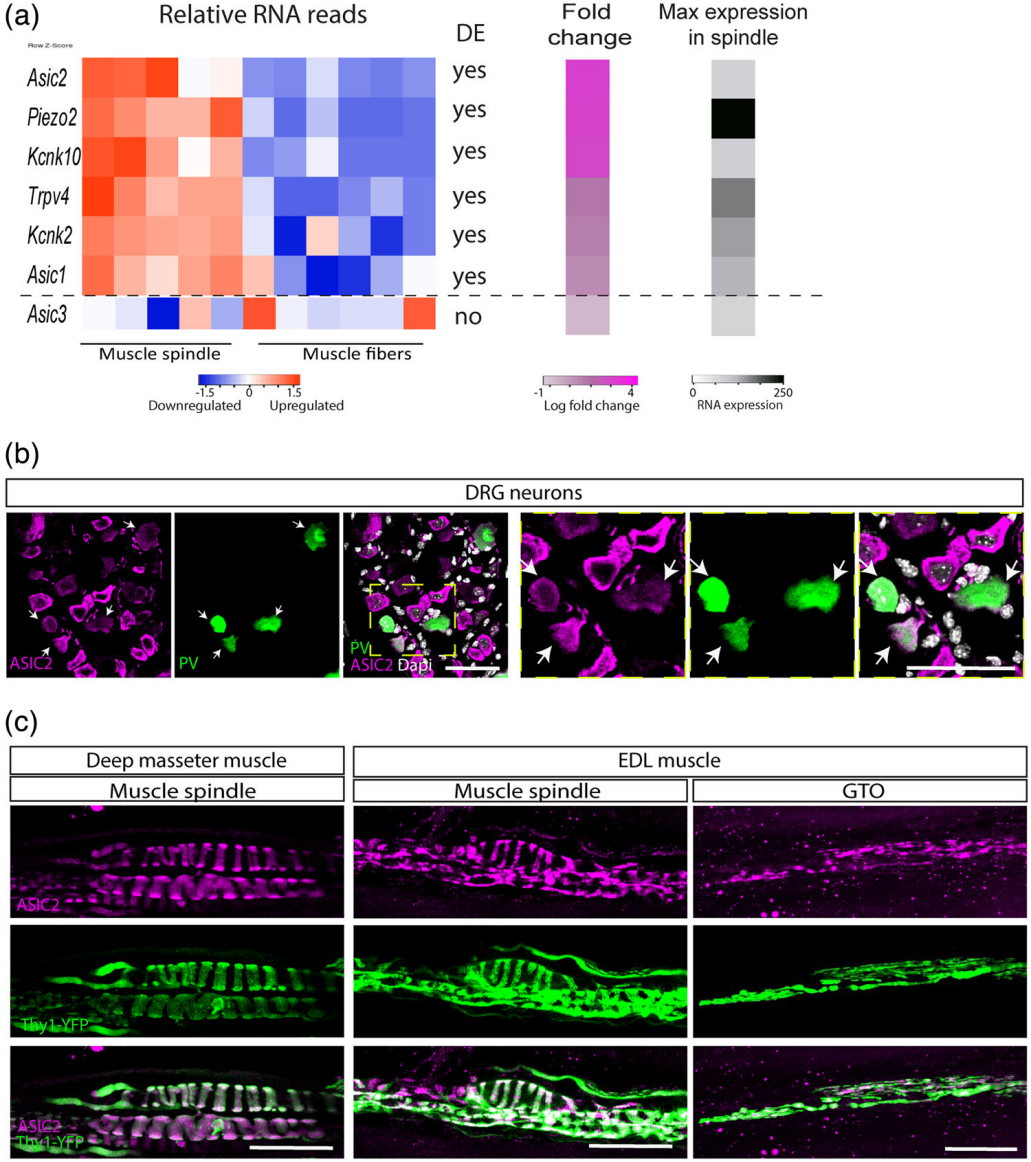
*Asic2* is expressed in proprioceptive sensory neuron terminals. (a) Left, heatmap showing the relative expression of mechanosensitive ion channels in muscle spindle samples compared with extrafusal fibres (Bornstein et al., 2023). The list to the right of the heatmap indicates whether the genes are differentially expressed (DE) between spindle and muscle samples. Right, colour bars showing the fold change in expression of each gene between the two samples (magenta) and the maximum expression of the gene in the muscle spindle samples (greyscale). (b) Confocal images of dorsal root ganglia (DRGs) from adult (>60 days old) *Pvalb^Cre^*; *Rosa26^tdTomato^* reporter mice (green) stained with antibody against ASIC2 (magenta). ASIC2 is expressed in *Pvalb* ^+^ DRG proprioceptive neurons (*n* = 50). Scale bars: 50 µm. (c) Confocal images of whole‐mount deep masseter (left) and extensor digitorum longus (EDL) muscle (right) from adult (>60 days old) *Thy1‐YFP* reporter mice (Feng et al., 2000) stained with antibodies against ASIC2 and GFP. ASIC2 is expressed in proprioceptive nerve endings innervating muscle spindles and Golgi tendon organs (GTOs) (*n* = 3 mice). Scale bars: 50 µm.

To identify potential mechanosensitive ion channels that mediate proprioceptive signalling, we examined our MS transcriptomic data (Bornstein et al., 2023). We found six channels that were differentially upregulated in MS samples compared with extrafusal muscle fibres (Figure 1a). Of those, *Asic2* was the most highly upregulated ion channel in our analysis (log fold change of 3.92; Figure 1a). In accordance with previous reports (Lin et al., 2016; Wu et al., 2021), we also detected expression of *Asic1* and *Asic3* (Figure 1a), suggesting that all three ASIC genes are expressed in muscle spindles. However, only *Asic1* and *Asic2* were upregulated in spindles, and the latter was the most differentially expressed mechanosensitive ion channel. Therefore, we proceeded to investigate *Asic2* involvement in proprioceptive function and skeletal regulation.

ASIC2 was previously shown to be expressed in adult rat muscle spindles in the deep lumbrical muscles (Simon et al., 2010). We therefore verified ASIC2 protein expression in proprioceptive neurons of mice by performing immunofluorescence staining. We first analysed ASIC2 expression in DRG neurons and found it to be widely expressed in sensory neurons (Figure 1b). To recognize proprioceptive neurons, we marked them genetically by crossing *Pvalb^Cre^* deleter mice with a *Rosa26^tdTomato^* reporter line (Hippenmeyer et al., 2005; Madisen et al., 2010). We detected ASIC2 expression in all parvalbumin‐positive neurons (*n* = 50; Figure 1b), confirming ASIC2 expression in proprioceptive neurons. Next, we performed whole‐mount immunofluorescence staining of the deep masseter muscle. We detected ASIC2 protein expression in the annulospiral endings of proprioceptive neurons innervating the central region of muscle spindles (Figure 1c). We confirmed the co‐localization of ASIC2 with the sensory nerve terminals by analysing the EDL muscle, where we detected ASIC2 expression in MSs and GTOs (Figure 1c).

### 3.2 Impaired performance of proprioception‐related tasks in *Asic2* KO mice

**FIGURE 2:**
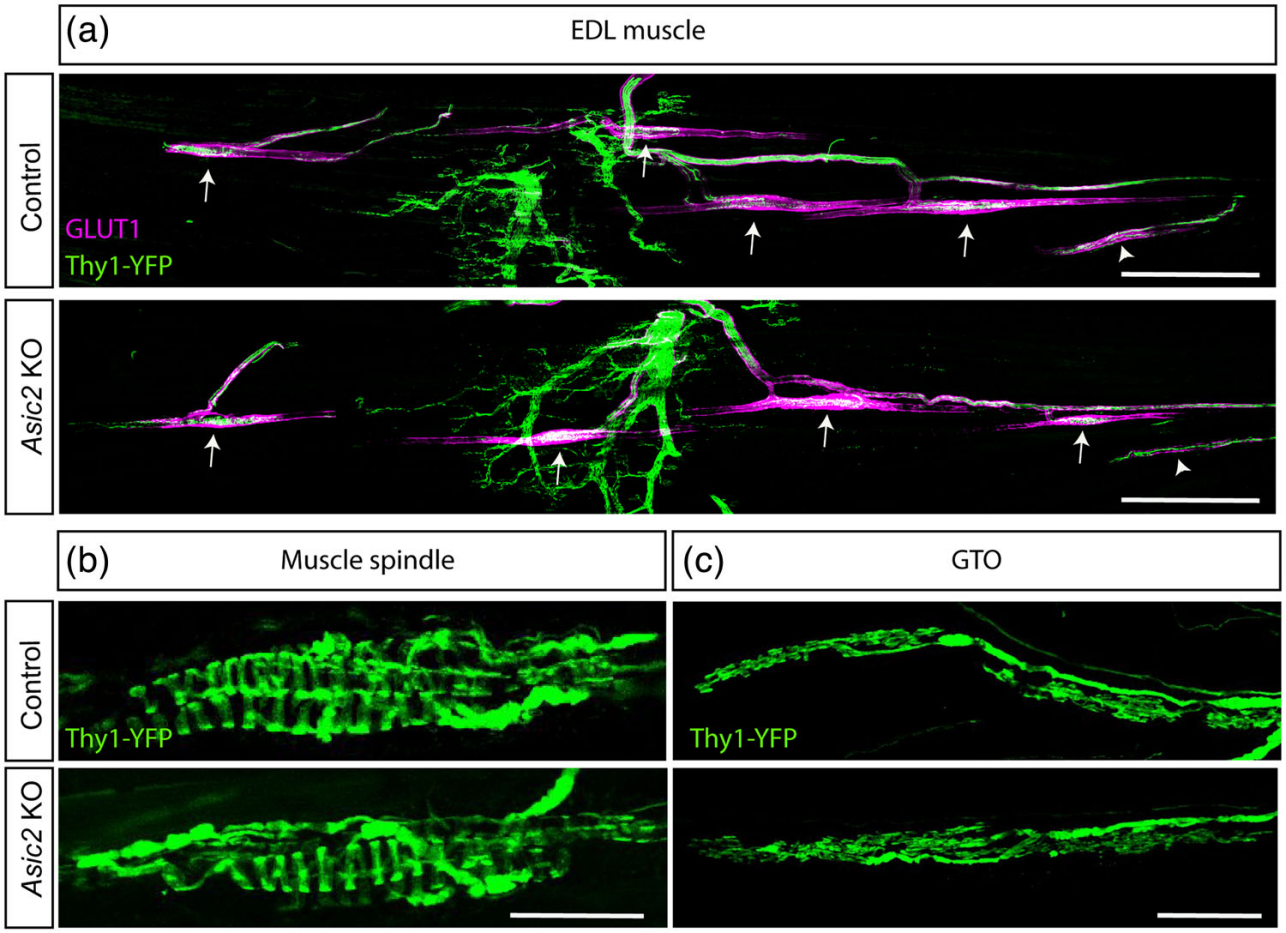
The morphology of muscle spindle and Golgi tendon organ (GTO) proprioceptive neurons is unchanged in *Asic2* knockout (KO) mice. (a–c) Confocal images of whole‐mount extensor digitorum longus (EDL) muscle taken from control mice (top) and *Asic2* KO mice (bottom) expressing *Thy1‐YFP*. The number of proprioceptors, marked by GLUT1 (a; magenta) and the morphology of muscle spindles (b) and GTOs (c) is similar in *Asic2* KO and control mice. Arrows indicate muscle spindles and arrowheads indicate GTOs. *n* = 5 in each group. Scale bars: 500 µm (a); 50 µm (b,c).

Previous studies of *Asic2* KO mice have implicated ASIC2 in the neurosensory mechanotransduction of touch (Price et al., 2000), the baroreceptive reflex (Lu et al., 2009), gastrointestinal sensing (Page et al., 2005) and pressure‐induced constriction in the middle cerebral arteries (Gannon et al., 2008). To determine whether ASIC2 also plays a role in proprioceptive function, we first examined the morphology of proprioceptive sensory neurons in *Asic2* KO mice (Price et al., 2000). To visualize MSs and GTOs, we crossed these mice with *Thy1‐YFP* reporter mice. Whole‐mount imaging revealed similar numbers and morphologies of proprioceptors in control and *Asic2* KO mice (Figure 2).

**FIGURE 3:**
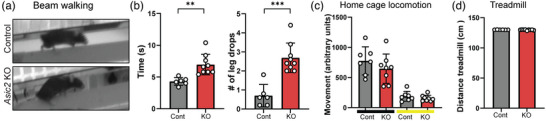
*Asic2* knockout (KO) mice display defects in performance of proprioception‐related tasks. (a,b) Beam walking test. Mice were trained to walk on a beam 6 mm wide and 50 cm long. The number of slips and the duration of crossing were measured in *Asic2* KO and control (cont) mice (*n*_KO_ = 9, *n*_Control_ = 8; time, *P* = 0.0022; leg‐drop, *P* = 0.00014; Student's two‐tailed *t*‐test), data are represented as the mean ± SD; each dot represents one mouse. (c) A home cage locomotion test was performed to measure locomotor abilities of *Asic2* KO and control mice in a single cage (*n*_KO_ = 8, *n*_Control_ = 7). No significant differences between the groups were found in either light (yellow line, *P* = 0.31) or dark (black line, *P* = 0.35) phases, as determined by Student's two‐tailed *t*‐test. Data are represented as the mean ± SD; each dot represents one mouse. (d) Treadmill test. Mice were trained to walk on a treadmill to avoid an electrical shock. The distance the mice travelled during the experiment was measured. *Asic2* KO mice travelled a similar distance to control mice (*n*_KO_ = 9, *n*_Control_ = 6; *P* = 0.51, Student's two‐tailed *t*‐test); data are represented as the mean ± SD; each dot represents one mouse.

We next examined the possible function of ASIC2 in the proprioceptive system by performing locomotion and coordination tasks. We first assessed coordination of *Asic2* KO and WT mice using the beam walking test (Brooks & Dunnett, 2009). *Asic2* KO mice needed significantly more time to cross the beam and displayed significantly more leg drops than their controls (Figure 3a,b), suggesting that ASIC2 is required for performance of proprioception‐related tasks. To rule out the possibility of a general effect on locomotion, we preformed home cage locomotion (Figure 3c) and treadmill (Figure 3d) tests (Brooks & Dunnett, 2009). We found similar locomotor abilities in *Asic2* KO and control mice, supporting our hypothesis that ASIC2 is important specifically for motor coordination.

**FIGURE 4:**
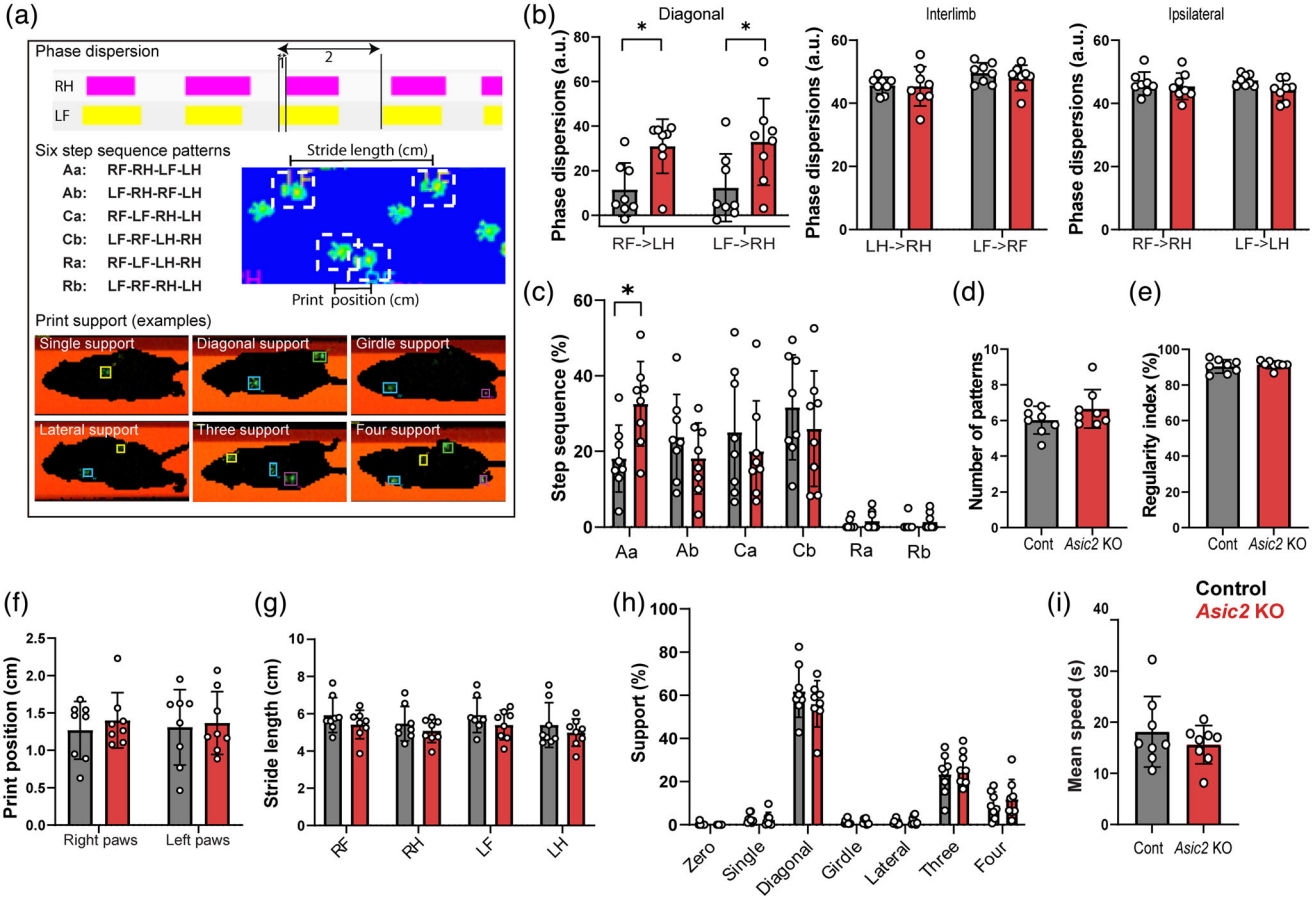
*Asic2* knockout (KO) mice display interlimb coordination defects. (a) Examples of CatWalk parameters indicating interlimb coordination. Phase dispersion describes the temporal relationship between placements of two paws. It measures the time interval (1) between the initial contact of an ‘anchor’ paw [here, left forelimb paw (LF)] and that of a ‘target’ paw [right hindlimb (RH)] as a percentage of the duration of a complete step cycle of the anchor paw (2). Step sequence pattern is the sequence of paw placement; the six most common patterns are presented. Step sequence categories: Aa, alternative a; Ab, alternative b; Ca, cruciate a; Cb cruciate b; Ra, rotary a; Rb, rotary b. Stride length is the distance between successive placements of the same paw, whereas print position is the distance between successive placements of different paws. Print support is the number and position of paws supporting the animal in each step. Key: LF, left forelimb (yellow); LH, left hindlimb (green); RF, right forelimb (blue); RH, right hindlimb (magenta). (b–i) Gait analysis using the CatWalk system. *Asic2* KO mice had a higher dispersion score in the diagonal phase (b; diagonal RF–LH, *P* = 0.012; diagonal LF–RH, *P* = 0.034; interlimb RF–RH, *P* = 0.667; interlimb LF–LH, *P* = 0.0801; ipsilateral LH–RH, *P* = 0.930; ipsilateral LF–RF, *P* = 0.857) and used the Aa step sequence pattern more frequently (c; Aa, *P* = 0.040; Ab, *P* = 0.862; Ca = 0.914; Cb = 0.866, Ra > 0.999, Rb > 0.999). No difference was found between *Asic2* KO mice and control mice in the number of patterns used (d; *P* = 0.202), regularity index (e; *P* = 0.566), print position (f; right paws, *P* = 0.789; left paws, *P* = 0.956), stride length (g; RF, *P* = 0.684; RH, *P* = 0.857; LF, *P* = 0.653; LH, *P* = 0.836), support (h; zero, *P* = 0.999; single, *P* = 0.999; diagonal, *P* = 0.385; girdle, *P* = 0.999; lateral, *P* = 0.999; three, *P* = 0.953; four, *P* = 0.961) or speed (i; *P* = 0.379) parameters [*P* > 0.05, ANOVA multiple comparison test in (b–h), Student's two‐tailed *t*‐test in (i)]. *n* = 8 in each group; data are represented as the mean ± SD; each dot represents one mouse.

To characterize coordination deficits associated with loss of *Asic2* further, we used the CatWalk system to analyse gait parameters in *Asic2* KO and control mice. We focused on parameters that were shown to reflect coordination abilities accurately, including interlimb parameters such as phase dispersion, step sequence, stride length, print position and support (Pitzer et al., 2021; Figure 4a). *Asic2* KO mice displayed a significant increase in dispersion score in the diagonal phase (i.e., right forelimb vs. left hindlimb paws and vice versa; Figure 4b), but not in ipsilateral or interlimb scores, indicating that the interval between placement of two diagonal paws is longer. Additionally, step sequence analysis showed that *Asic2* KO mice use the Aa alternate pattern of walking (right fore–right hind–left fore–left hind) significantly more than control mice (Figure 4c). No difference was found between genotypes in the number of patterns used, regularity index, stride length, print position or support parameters (Figure 4d–h), indicating a restricted effect on interlimb coordination between diagonal paws. Additionally, *Asic2* KO mice moved at a similar speed to control mice (Figure 3i), supporting our observation that *Asic2* mutation does not affect general motor abilities, but rather movement coordination specifically.

### 3.3 Muscle spindle response to stretch is different in *Asic2* KO mice

**FIGURE 5:**
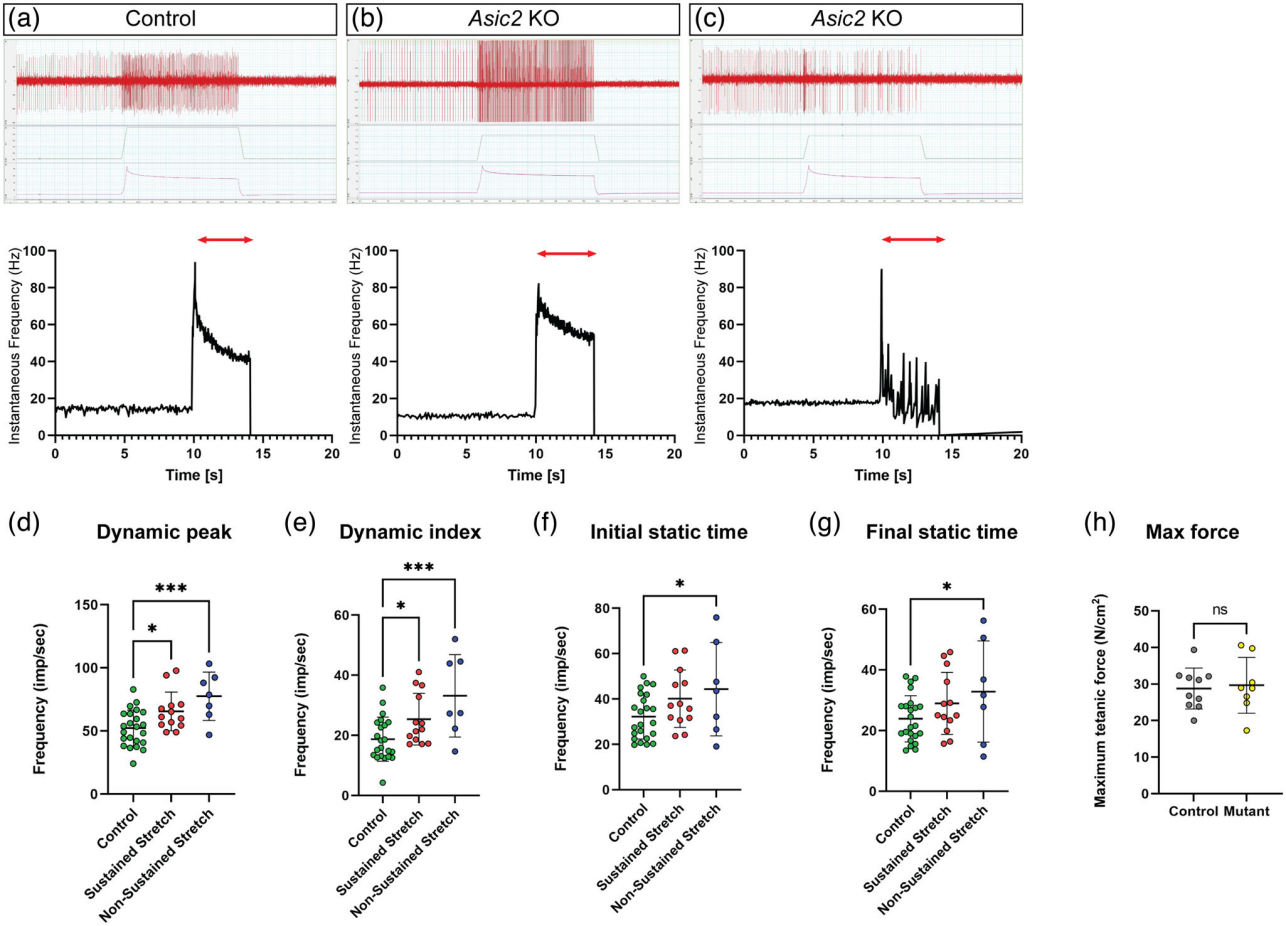
Muscle spindle afferent response to stretch is altered in *Asic2* knockout (KO) mice. (a–c) Top, the responses of muscle spindles to a 7.5% stretch from resting length (*L*_0_) in control mice (a) and *Asic2* KO mice (b,c). Muscle spindle recordings from *Asic2* KO mice show sustained responses (b) and non‐sustained responses (c). Bottom, the instantaneous frequency of the responses shown in the top panel. Red arrows indicate the duration of stretch. (d–g) Comparison of the instantaneous frequency of the dynamic peak (d; control vs. sustained stretch, *P* = 0.0181; control vs. non‐sustained stretch, *P* = 0.0005), dynamic index (e; control vs. sustained stretch, *P* = 0.0415; control vs. non‐sustained stretch, *P* = 0.0007), initial static time (f; control vs. sustained stretch, *P* = 0.0852; control vs. non‐sustained stretch, *P* = 0.0358) and final static time (g; control vs. sustained stretch, *P* = 0.1612; control vs. non‐sustained stretch, *P* = 0.0493) during 5% *L*_0_ ramp‐and‐hold stretch between control mice (green dots) and *Asic2* KO mice with a sustained response to stretch (red dots) and *Asic2* KO mice with a non‐sustained response to stretch (blue dots). *n*_Control_ = 23, *n*_KO_ = 20; ordinary one‐way ANOVA with Fisher's LSD. (h) Comparison of the maximum tetanic force of the extensor digitorum longus (EDL) muscle between control mice (grey dots) and *Asic2* KO mice (yellow dots) shows no significant difference (*n*_KO_ = 8, *n*_Control_ = 10; *P* = 0.77, Student's two‐tailed *t*‐test).

Given that *Asic2* KO mice displayed deficits in proprioception‐related behavioural tasks, we next analysed the effect of *Asic2* deletion on MS function. Using an ex vivo electrophysiology preparation, we compared muscle spindle afferent firing during stretch between control and *Asic2* KO mice. We observed heterogeneous firing rates in all muscle spindles from mutant mice (*n* = 4) in comparison to heterozygous (*n* = 3) and WT animals (*n* = 2) (pooled as the control group). However, the abnormalities were very variable, even in MSs from the same muscle. The responses to stretch in *Asic2* KO mice could be categorized qualitatively into those that showed sustained firing in response to stretch (Figure 5b) or those that ceased firing for short moments during the hold phase of a ramp‐and‐hold stretch (Figure 5c).

To study the response to stretch in more detail, ramp‐and‐hold stretches of three different magnitudes (2.5, 5.0 and 7.5% of resting length, *L*_0_) were applied, and the dynamic peak (DP), dynamic index (DI), initial static time (IST) and final static time (FST) (Kröger & Watkins, 2021) were compared between *Asic2* KO and control mice (Figure 5d–g; 5% *L*_0_ ramps are shown). The muscle spindles that exhibited a sustained stretch response (Figure 5d–g, red dots) had a significantly increased instantaneous frequency over the dynamic peak (Figure 5d) and dynamic index (Figure 5e), while the muscle spindles that exhibited a non‐sustained stretch response (Figure 5d–g, blue dots) had a significantly increased instantaneous frequency in all four parameter analysed (Figure 5d–g). In contrast, the maximum tetanic force (Figure 5h) was not significantly different between control and *Asic2* KO mice, indicating that the altered spindle responses were not attributable to impaired muscle health. Taken together, these results indicate that ASIC2 is required for modulation of proprioceptive afferents in response to stretch.

### 3.4 *Asic2* loss of function results in skeletal malalignment

**FIGURE 6:**
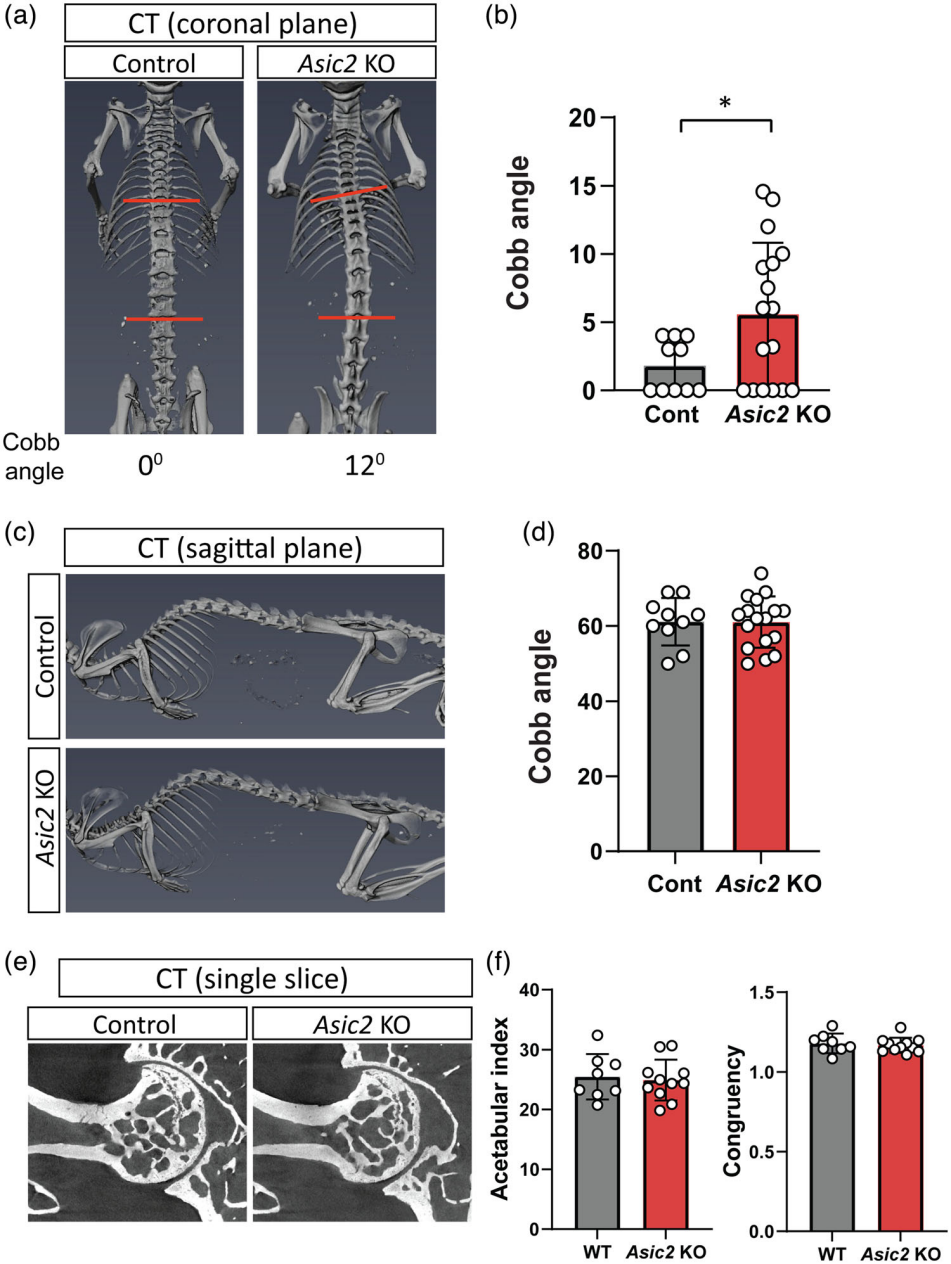
Loss of *Asic2* results in spine malalignment but not in altered hip morphology. (a) In vivo CT scans of spines of control and *Asic2* knockout (KO) mice aged >90 days, showing mild scoliosis (coronal plane) in the mutants. (b) Quantification of the Cobb angle measured in control (*n* = 10) and *Asic2* KO (*n* = 17) mice (*P* = 0.039, Student's two‐tailed *t*‐test). (c) In vivo CT scans of the spines of control and *Asic2* KO mice aged >90 days, showing no difference in kyphosis (sagittal plane) between mutants and control animals. (d) Quantification of the Cobb angle measured for control (*n* = 10) and *Asic2* KO (*n* = 17) mice (*P* = 0.97, Student's two‐tailed *t*‐test). (e) Ex vivo CT scans of hip joints of control and *Asic2* KO mice aged >90 days, showing no signs of dysplasia in mutants. (f) Graphs showing the acetabular index and congruency index (upper lips) in control (*n* = 8) and *Asic2* KO mice (*n* = 10). No significant differences were found in either measurement (*P* = 0.75 and *P* = 0.65, respectively, Student's two‐tailed *t*‐test). Data in (b,d,f) are represented as the mean ± SD; each dot represents one mouse.

Previously, we have demonstrated that the proprioceptive system is necessary to maintain skeletal integrity (Assaraf et al., 2020; Blecher, Krief, Galili, Assaraf, et al., 2017; Blecher, Krief, Galili, Biton, et al., 2017). Having found that ASIC2 functions to mediate proprioceptive sensing, we proceeded to assess whether loss of *Asic2* would have an effect on the skeleton. For that, we compared spinal alignment between control and *Asic2* KO mice by using CT to determine the level of scoliosis and kyphosis in these mice. Scoliosis was defined as a lateral curve of the spine >10° in the coronal plane and kyphosis as excessive angulation of the spine in the sagittal plane compared with control animals. The results showed that 30% (5 or 17) of the *Asic2* KO mice exhibited mild scoliosis (Figure 6a,b), measured as a Cobb angle ranging from 10 to 15°. In comparison, none of the control mice had a curve of >10°. Interestingly, the scoliotic phenotype of the *Asic2* KO mice was not accompanied by kyphosis (Figure 6c,d), indicating that *Asic2* ablation affects only one plane of spine alignment.

Proprioception deficits also affected hip joint morphology, resulting in a shallow acetabulum and loss of joint congruency (Assaraf et al., 2020). Therefore, we analysed the hip joints of *Asic2* KO mice for features of hip dysplasia. However, micro‐CT images of hip joints from *Asic2* KO mice and control littermates showed similar morphologies and no signs of hip dysplasia (Figure 6e), suggesting that loss of *Asic2* does not affect hip joint morphology.

Collectively, our results show that *Asic2* is expressed by proprioceptive neurons and plays important roles in mediating proprioceptive sensing, motor coordination and spine alignment.

## 4 DISCUSSION

In this work, we showed that the mechanosensitive ion channel ASIC2 is expressed by proprioceptive neurons innervating MSs and GTOs. We then demonstrated its function in mediating proprioceptive sensing, motor coordination‐related tasks and spine alignment, thus showing that ASIC2 is an important mediator of proprioceptive function.

ASIC2 was shown to be involved in mechanosensing of baroreceptive neurons (Lu et al., 2009) and of low‐threshold cutaneous neurons (Price et al., 2000). Here, we have shown that ASIC2 contributes to proprioceptive sensing, as *Asic2* KO mice displayed an altered MS response to stretch and impaired performance of coordination‐related tasks. These phenotypes are similar to proprioception defects observed in *Asic3* loss‐of‐function mice (Lin et al., 2016). Given that functional ASIC channels are homotrimers or heterotrimers assembled from three subunits (Kang et al., 2012), this phenotypic similarity might indicate redundancy between the different channels. However, given that ASICs can form heterotrimeric complexes, we cannot rule out the possibility that ASIC2 and ASIC3 function together to regulate proprioception. Further investigation using *Asic2* and *Asic3* double‐KO mice is needed to decide between these options.

Interestingly, we noticed heterogeneous afferent firing patterns in response to stretch in *Asic2* KO mice. A similar effect of opposite firing responses was also observed in stretch‐evoked recordings of *Asic3* KO mice (Lin et al., 2016). These phenotypes could be explained by the fact that different combinations of ASIC subunits exhibit different electrophysiological properties (Cheng et al., 2018; Jasti et al., 2007). Thus, differences in ASIC composition between proprioceptive neurons would result in variation in sensitivities and ranges of firing responses to stretch. Indeed, single‐cell RNA‐seq analysis of proprioceptive neurons identified the expression of *Asic1* and *Asic2* in all subtypes of proprioceptive neurons, whereas *Asic3* was expressed in only a few subtypes (Wu et al., 2021), showing that different ASIC subunits are expressed by different individual neurons. Furthermore, the identification of different subtypes of proprioceptive neurons (Oliver et al., 2021; Wu et al., 2021) suggests that different proprioceptors have different functions. Thus, the combinatory expression of ASIC subunits suggests a mechanism for diversity in the sensing abilities of proprioceptive neurons. However, it is still unclear how knockout of one ASIC gene affects the expression of different ASIC subunits in different neurons and how the ASIC subunit compositions translate into different proprioceptive signals.

The connection between the proprioceptive system and the skeleton was recently established (Bornstein et al., 2021), but most of the molecular components of this system that are involved in skeletal pathologies are unknown. Here, we have identified the ion channel ASIC2 as a regulator of skeletal integrity. Interestingly, we found that *Asic2* ablation affects only the lateral curve of the spine, causing scoliosis, without causing kyphosis or affecting hip joint morphology. To our knowledge, this is the first proprioception regulatory gene whose ablation selectively affects only one plane of skeletal alignment. This specific phenotype is consistent with previous observations that the severity of the skeletal phenotype is correlated with the severity of the proprioceptive defect. Specifically, *Runx3* KO mice, which lack functional proprioceptive neurons, display much stronger skeletal phenotypes than *Egr3* KO mice, which lack muscle spindles but not GTOs (Blecher, Krief, Galili, Biton et al., 2017). Thus, one explanation for the observed scoliosis is that the lateral curve of the spine is the most sensitive to proprioception defects. Alternatively, the specific effect of *Asic2* deletion might imply that proprioception regulates various aspects of skeletal integrity via different mechanisms. To decide between these two options, it will be necessary to evaluate the skeletal phenotypes caused by deletion of other genes that affect proprioception mildly. Thus, to gain a better understanding of the regulatory role of the proprioceptive system in skeletal biology, it is necessary to identify and study additional molecular players that mediate proprioception.

One limitation of our study is that we used a conventional KO approach to delete *Asic2*. Given that ASIC2 expression in the DRG is not limited to proprioceptive neurons (Cheng et al., 2018; García‐Añoveros et al., 2001; Price et al., 2000), we could not determine its cell‐autonomous effects in different tissues. This question should be addressed in future studies by applying tissue‐specific *Asic2* knockout.

Pervious works showed that *Piezo2* loss of function leads to a complete loss of proprioceptive neuron mechanotransduction and to severe abnormalities of the spine and hips (Assaraf et al., 2020; Woo et al., 2015). Interestingly, the *Asic2* loss‐of‐function mice displayed milder phenotypes in both mechanotransduction and skeletal alignment. This might suggest that PIEZO2 is necessary to generate signals in proprioceptive neurons, whereas ASIC2 functions to modulate the signal.

Overall, here we have identified the mechanosensitive ion channel ASIC2 as another molecule that mediates proprioceptive sensing and skeletal alignment. The effect of ASIC2 on the skeleton also reveals the complexity of the regulatory interactions between the proprioceptive system and skeletal development.

## AUTHOR CONTRIBUTIONS

Experiments were conducted in the laboratories of Stephan Kröger and Elazar Zelzer. Conception or design of the work: Bavat Bornstein and Elazar Zelzer. Acquisition, analysis or interpretation of data for the work: Bavat Bornstein, Bridgette Watkins, Fabian S. Passini, Ronen Blecher, Eran Assaraf, XiaoMeng Sui, Vlad Brumfeld, Michael Tsoory, Stephan Kröger and Elazar Zelzer. Drafting of the work or revising it critically for important intellectual content: Bavat Bornstein, Bridgette Watkins, Fabian S. Passini, Ronen Blecher, Eran Assaraf, XiaoMeng Sui, Vlad Brumfeld, Michael Tsoory, Stephan Kröger and Elazar Zelzer. All authors approved the final version of the manuscript and agree to be accountable for all aspects of the work in ensuring that questions related to the accuracy or integrity of any part of the work are appropriately investigated and resolved. All persons designated as authors qualify for authorship, and all those who qualify for authorship are listed.

## CONFLICT OF INTEREST

None declared.

## Supporting information



Statistical Summary Document

## Data Availability

All data that support the findings of this study are available from the corresponding authors upon request.
